# Artificial Intelligence in Biomedical Engineering and Its Influence on Healthcare Structure: Current and Future Prospects

**DOI:** 10.3390/bioengineering12020163

**Published:** 2025-02-08

**Authors:** Divya Tripathi, Kasturee Hajra, Aditya Mulukutla, Romi Shreshtha, Dipak Maity

**Affiliations:** 1 School of Health Sciences, University of Petroleum and Energy Studies, Dehradun 248007, Uttarakhand, India; divya.tripathi@ddn.upes.ac.in (D.T.);; 2 School of Public Health, SRM Medical College, Chennai 603203, Tamil Nadu, India; 3 Integrated Nanosystems Development Institute, Indiana University Indianapolis, Indianapolis, IN 46202, USA; 4 Department of Chemistry and Chemical Biology, Indiana University Indianapolis, Indianapolis, IN 46202, USA

**Keywords:** artificial intelligence, biomedical engineering, diagnostics, biomarker, biosensors, imaging, biomedical devices

## Abstract

Artificial intelligence (AI) is a growing area of computer science that combines technologies with data science to develop intelligent, highly computation-able systems. Its ability to automatically analyze and query huge sets of data has rendered it essential to many fields such as healthcare. This article introduces you to artificial intelligence, how it works, and what its central role in biomedical engineering is. It brings to light new developments in medical science, why it is being applied in biomedicine, key problems in computer vision and AI, medical applications, diagnostics, and live health monitoring. This paper starts with an introduction to artificial intelligence and its major subfields before moving into how AI is revolutionizing healthcare technology. There is a lot of emphasis on how it will transform biomedical engineering through the use of AI-based devices like biosensors. Not only can these machines detect abnormalities in a patient’s physiology, but they also allow for chronic health tracking. Further, this review also provides an overview of the trends of AI-enabled healthcare technologies and concludes that the adoption of artificial intelligence in healthcare will be very high. The most promising are in diagnostics, with highly accurate, non-invasive diagnostics such as advanced imaging and vocal biomarker analyzers leading medicine into the future.

## 1. Introduction

A form of computer science, artificial intelligence (AI) is computational intelligence that can see and process information in a way that solves problems and makes decisions, often beyond the capabilities of human and non-human minds [1]. The replication of human intellect in robots built to carry out tasks that normally require cognitive abilities like learning, reasoning, and decision-making is known as AI. AI has transformed the life sciences industry and created new possibilities and continued improvement. AI is a system that includes two main parts, software and hardware. Machine learning (ML), Natural Language Processing (NLP), and deep learning (DL) are just a few of the subfields that fall under AI, and each has its own unique approaches and uses. ML algorithms allow systems to identify patterns in data and make predictions or decisions without being explicitly programmed, using approaches like Supervised Learning, Unsupervised Learning, and reinforcement learning. Deep learning, a branch of machine learning, uses artificial neural networks, especially multilayered ones, to draw meaningful insights from raw data. For instance, convolutional neural networks (CNNs) are used for image recognition, while recurrent neural networks (RNNs) are great for understanding sequences in data, like time series or language [2].

In healthcare, deep-learning algorithms have improved diagnosis and procedures by a wide margin. Pathology and medical imaging are just two applications of AI used to better analyze, process, and reconstruct images, thereby achieving better disease detection [3]. The other notable use of AI is in understanding genome information that can be vital for healthcare professionals studying genetic disorders and drug resistance [3]. Also, ML and CNNs have been used in Protein Structure Prediction, which is a key task in modeling protein folding and structure in drug design [4].

In epidemiology and public health, AI assists with disease transmission predictions and implements favorable control measures [5]. Geospatial AI improves data resolution and processing to better understand environmental drivers of disease propagation and surveillance [6]. The administration of the hospital also benefits from AI, with pharmaceutical logistics, staff scheduling, and space allocation being simplified [7]. Companies such as IBM Watson Health offer doctors AI-enabled instruments so that they can give the individual the medical attention they need based on their history and genes. These are solutions that save diagnostic costs and unneeded tests [8].

**Figure 1:**
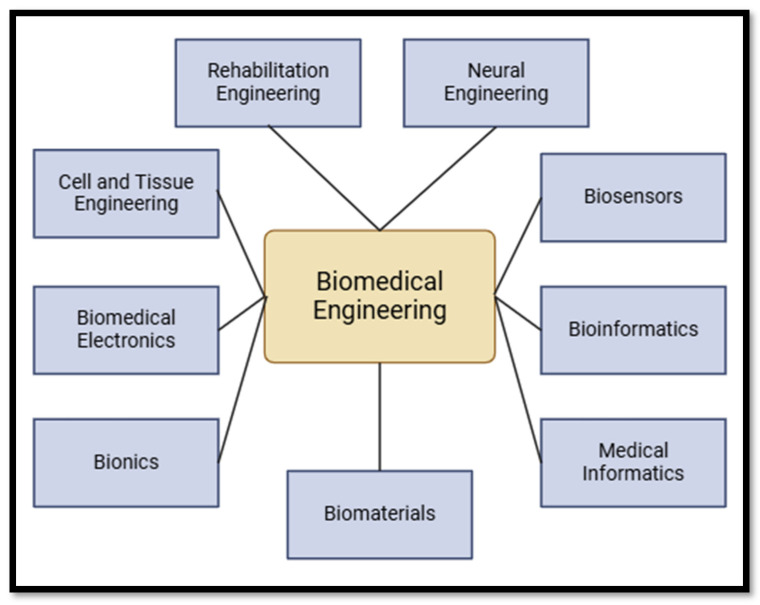
Biomedical engineering: a miscellaneous and multidisciplinary field that conglomerates principles from engineering, biology, medicine, and other sciences to develop innovative solutions for healthcare and medical challenges.

Secondly, AI-enabled household and wearable devices (known as “Telehealth”) have widened access to care for elderly and differently abled people. They facilitate remote monitoring of health metrics, from dietary consumption to mental health [9]. The same technologies are present in the area of fitness and sports, known as ‘Smart Fitness’, where watches track bodily activity and health metrics [10]. Figure 1 shows multiple domains of biomedical engineering in healthcare.

AI is becoming a cornerstone of biomedical engineering by enhancing diagnostic capabilities, personalizing treatment strategies, improving biomedical device performance, and optimizing healthcare systems [11]. For instance, in medical imaging, it helps by improving the accuracy of diagnoses in radiology through advanced image processing techniques, which can identify patterns and anomalies that might elude human eyes. An AI model developed by Google Health was trained on a large dataset of mammograms and was evaluated in a real-world clinical setting, which outperformed human radiologists with fewer false positives and false negatives. This model was developed to assist radiologists in detecting breast cancer from mammograms [12]. However, the physicians working with these models and AI systems must understand how they work and should be able to interpret predictions, assess performance, and compare models to identify the best fitted model for their clinical applications [13]. The CAM (Class Activation Mapping) model enhances medical imaging by offering clear visual explanations for AI decisions by highlighting key regions that have influenced AI outcomes [14]. Thereby, the collaboration between AI and biomedical engineering is shaping the future of healthcare and improving patient outcomes globally.

Research on AI in healthcare is very much underway. There are examples of using AI to discover three-dimensional biomolecules like proteins in the biotechnology industry. The production of drugs and chemicals can also be optimized with AI’s drug and chemical manufacturing. AI can assist in drug discovery, detection, and targeting structures in microbiota engineering, ushering in an exciting new era of immunology and pharmacology [15]. AI is also useful in pathogen detection and cluster detection of antimicrobial resistant pathogens, which is a key domain for the world’s health [16]. Artificial intelligence, fundamentally, involves the replication of human intelligence within machines, enabling them to perform tasks that traditionally require human cognition and decision-making capabilities.

Biomedical engineering is evolving due to AI, which enables the development of innovative and effective solutions to complex healthcare problems. But integrating AI into this broad, interdisciplinary subject necessitates a concentrated investigation to pinpoint its precise contributions and tackle related issues. This paper focuses on the use of AI in biomedical engineering, including fields such as medical imaging, diagnostics, disease surveillance, and disease outbreak detection, which has become a crucial field where cutting-edge algorithms are transforming patient outcomes, diagnosis, and treatment planning. By combining developments in this specialized field and demonstrating its applicability through current technological and healthcare improvements, this paper seeks to close the gap left by more comprehensive evaluations that attempt to cover all AI applications in biomedical engineering. The intention is to open the door for focused breakthroughs in this subject by offering scholars and practitioners practical insights.

## 2. Artificial Intelligence

AI is a multidisciplinary field encompassing computer and data science aimed at developing synthetic intelligence in machines that mimic human capabilities such as sensory perception, recognition, reasoning, communication, and learning [17]. Early milestones in AI include programs like the Logical Theory Machine, created by Newell and Simon [18], and the logic programming system developed by Christopher Strachey in the 1950s. These pioneering efforts inspired generations of researchers to explore the vast potential AI holds for various domains.

The study of AI is broadly categorized into six core areas: machine learning, deep learning, neural networks, cognitive computing, Natural Language Processing, and computer vision.

**Figure 2:**
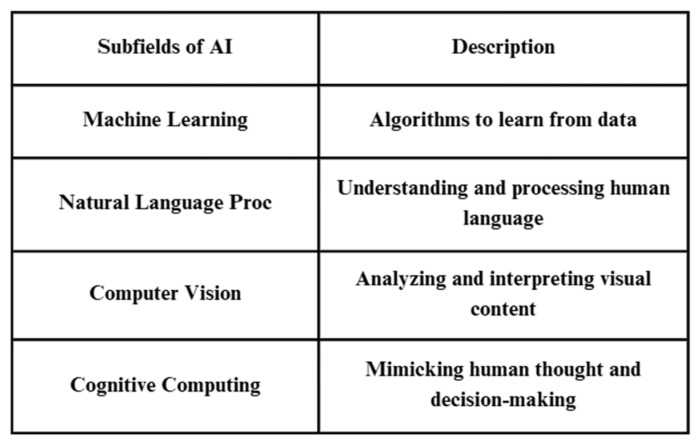
Subfields of AI and their description. AI refers to the simulation of human intelligence in machines that can perform tasks that typically require human intelligence. AI encompasses a wide range of techniques and technologies, each with their own components.

AI-powered robotic systems have revolutionized healthcare systems by assisting surgeons in invasive surgeries and minimizing complications. Moreover, AI-driven prosthetics and exoskeletons can assist patients with spinal cord injuries in walking and executing less extensive movements. These rehabilitation robots can improve their functionality by learning and adapting to the user’s movements, which can provide personalized rehabilitation solutions [19,20]. Subfields of AI have been summarized in Figure 2. 

## 3. Machine Learning

Machine learning is the field which tries to make the machine efficient and can work with any amount of data. It allows computers to work independently of explicit code to read and process information [21]. Because of this flexibility, machine learning is today a common tool in applications that have large-scale complex data. The machine programming techniques that scientists use include Supervised Learning (SL), Unsupervised Learning (UL), and Semi-Supervised Learning (SSL).

Machines are programmed in Supervised Learning to make predictions from given input data but need prior instructions from the programmers. The popular use of Linear Support Vector Machines (SVMs) in biology [22] is a case in point. Unsupervised Learning, on the other hand, does not use any previous programming to guide the machine. Rather, the machine is autonomous, and it chooses to maximize good outcomes as it sees fit. These are algorithms like K-means Clustering, Principal Component Analysis, etc. [23].

Semi-Supervised Learning is both Supervised and Unsupervised Learning. It relies on self-training and Transductive Support Vector Machines (TSVMs) to reach a middle ground between guided and autonomous learning.

ML algorithms are employed in diagnostic tools to predict disease outcomes by analyzing complex datasets from electronic health records and patient histories. Moreover, ML models are used to identify patterns in genetic information, including different forms of mutations, which helps in the development of targeted therapies for diseases including cancer [24].

## 4. Natural Language Processing

Natural Language Processing (NLP) is a mix of technology and language to comprehend speech and non-speech. Through statistical and probabilistic techniques, NLP guesses the words or speech and pinpoints context [25]. Developments in this area have resulted in AI-powered chatbots such as “Siri” and “Alexa”.

NLP is also being applied to the accessibility and functionality of electronic health record (EHR) platforms in healthcare [26]. They use patient information, like symptoms from across clinical areas, to mine, categorize, analyze, and deliver data appropriately [27]. In the biomedical field, NLP is used in computational life sciences and medical science research as a tool of bioinformatics [28]. For instance, NLP algorithms can be applied in the processing and understanding of vast amounts of biomedical literature and clinical notes and to obtain meaningful insights from unstructured text data, like identifying potential drug interactions [29]. Another application of NLP is in the design of telepresence robots, chatbots, and virtual assistants to provide patients with timely and accurate medical information, which enhances healthcare accessibility and patient engagement [30].

## 5. Neural Networks

Neural networks structure computational systems to mimic the processing patterns observed in biological organisms, including humans. Among the many types of neural networks, convolutional neural networks (CNNs) and artificial neural networks (ANNs) are the most notable. CNNs are primarily employed to solve problems by identifying patterns within input data, while ANNs are designed to tackle more complex challenges that require approximation and adaptive learning capabilities. Neural networks form the foundation of both machine learning and deep learning. Most machine learning methodologies utilize neural networks, whereas deep learning networks represent an advanced form, consisting of multiple interconnected neural network layers (three or more) working in tandem [28]. In healthcare, neural networks power a range of smart applications, including wireless body sensors [27], mobile health applications, accelerator chips [31], as well as tools for decision-making and disease prediction [32].

## 6. Deep Learning

Deep learning enables machines to comprehend abstract and complex concepts, such as social cues, traffic regulations, and human conversations. Notable deep learning frameworks include Deep Belief Networks and Deep Boltzmann Machines, both of which are built upon Restricted Boltzmann Machine (RBM) neural networks.

This field has played a critical role in advancing other domains. For instance, its integration with Computer Vision Technology has significantly enhanced capabilities such as facial and object recognition, position estimation, and imaging precision [33].

DL algorithms are particularly useful in detecting abnormalities in radiological images. For instance, image classification and segmentation tasks can be effectively performed with the aid of CNN, thereby facilitating early and accurate diagnosis [34].

## 7. Cognitive Computing

Cognitive computing, a subset of artificial intelligence, empowers computing systems to emulate human intelligence for problem-solving [35]. Unlike autonomous problem-solving, cognitive machines, primarily driven by AI, are designed to assist humans in performing tasks.

Advancements in this field have enabled devices to interpret and respond to human emotions [36], opening up commercial opportunities for AI applications in sectors like healthcare and hospitality. In healthcare, cognitive computing has facilitated the development of AI-based Clinical Decision Support Systems (CDSSs), which play a crucial role in aiding diagnosis, treatment [37], and surgical procedures [38].

## 8. Computer Vision

Computer vision focuses on improving a computer’s ability to identify and recognize visual images. By incorporating techniques from fields like deep learning and machine learning, it analyzes image data to achieve highly accurate results in detection and interpretation.

In biology, computer vision holds significant potential for advancing medical diagnostics. Its applications are particularly promising in microscopy, where it can be used to study cell morphology [39], detect pathogens such as malaria [40], and even identify microscopic cancers [41].

## 9. Advances in Artificial Intelligence in the Medical Sciences

The adoption of AI in medicine has brought about astronomical improvements in patient care, diagnostic and therapeutic tools, and personalized medicine. It is this kind of use of AI that continues to drive innovations in medical sciences as research scales to realize its potential in these areas.

Deep learning has also made diagnostic devices like Computer Tomography and Ultrasonography accurate, making it easier to understand the results and allowing for less invasive testing [42,43]. These innovations make patients easier to diagnose. Machine learning algorithms, for example, have identified biomarkers for disorders such as Acute Renal Failure (ARF), which allow for an early diagnosis and earlier treatment [44]. Further innovations come from the use of nanotechnology and AI with deep learning-powered biosensors for disease biomarkers. The “Hospital-on-a-Chip” model allows for early, even at-home, diagnosis [45].

In pharmacology, machine learning optimizes Computer–AI Synthesis Planning (CASP) to develop new medical formulations [38]. Additionally, these algorithms predict drug–drug interactions (DDIs) and combining multiple such algorithms has demonstrated superior accuracy compared to individual systems. This is particularly beneficial for managing complex disorders like osteoporosis and Paget’s disease [46].

AI also shows promise in augmenting patient care by assisting nursing professionals. Although current studies have not conclusively affirmed the integration of AI into nursing workflows [47], AI nurse assistants could help mitigate the effects of understaffing, a persistent challenge in recent years [48]. By sharing the workload, AI systems could potentially improve patient survival rates [49]. Moreover, efforts are underway to merge robotics with AI to create surgical assistants. While these systems are primarily limited to non-invasive procedures like laparoscopies [50,51,52], they raise ethical concerns regarding the privacy of patient data and the possibility of robots replacing surgeons, leading to hesitation among physicians about using AI in operating rooms [53]. Nonetheless, AI could alleviate the workload of surgeons, particularly in the post-COVID-19 healthcare environment.

Despite challenges in fully integrating AI into the responsibilities of doctors and nurses, its effectiveness in streamlining healthcare processes is evident. For example, a Natural Language Processing-enhanced conversational agent was successfully employed for post-operative follow-ups with orthopedic surgery patients, demonstrating performance comparable to that of human staff [54]. While such solutions may not be necessary for smaller clinics, they can be invaluable for large hospitals with heavy patient loads. AI has also shown promise in identifying Surgical Site Infections (SSIs), which, although rare in sterile environments, can lead to severe complications like sepsis. The immediate detection of SSIs can facilitate timely follow-up care and minimize surgery risks [55].

Furthermore, AI contributes to post-operative care beyond physical health by addressing psychological well-being. For instance, a study utilizing social media data, including user photos and metadata from Instagram, demonstrated that AI could identify depression with higher accuracy than unassisted practitioners [56]. This innovative application of computer vision and machine learning holds immense potential for improving mental health screening processes. Beyond depression, these tools could be applied to conditions such as Post-Traumatic Stress Disorder (PTSD), anxiety, postpartum depression, and psychosis.

**Figure 3:**
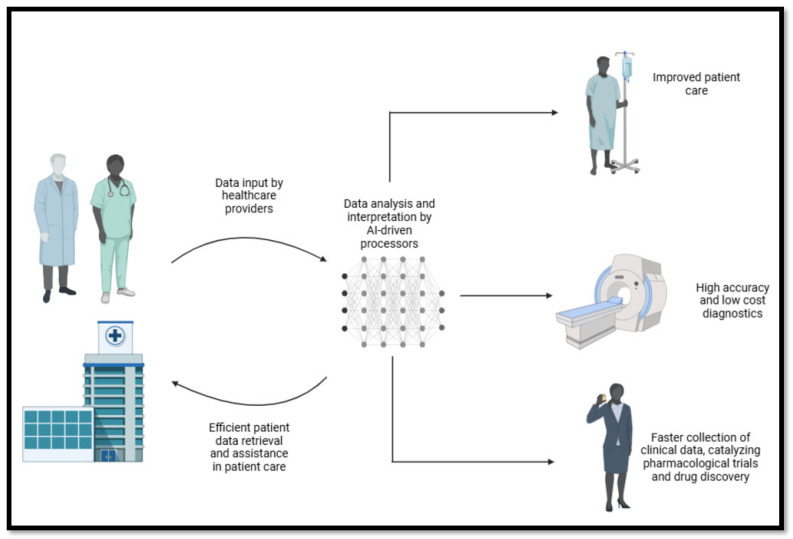
The various benefits of artificial intelligence Integration.

Figure 3 describes a simplified model by which AI-driven technology can enhance medical services provided by the healthcare industry. AI’s ability to analyze big data derived from various sources, including medical professionals and patient experiences, makes it an ideal tool for various purposes. For example, it can be used to improve patient care, optimize diagnostic results, and identify novel symptoms/complications/biomarkers, which are of significant interest in the development of pharmacological agents.

In conclusion, AI exhibits remarkable functionality, enabling its application across virtually all healthcare sectors. However, its integration with medicine remains at an intermediate stage and has yet to fully align with the immense potential AI offers. Advancing this integration requires further research, informed collaboration with medical professionals, and careful consideration of ethical implications. These steps are essential for unlocking the transformative capabilities of AI in healthcare. The advantages of integrating AI into medical practice are summarized in Figure 3.

## 10. Reasons for Using Artificial Intelligence in Biomedical Sciences

Late disease detection and the absence of early treatment and adequate healthcare facilities contribute significantly to complications and mortality. Accurate disease diagnosis often requires substantial time and testing, which can be prohibitively expensive, placing a financial strain on patients and their families [57]. Additionally, prescribed medications may not always be tailored to the patient’s specific needs or conditions. To address these challenges, machine learning (ML) and deep learning (DL) tools have been leveraged to develop cost-effective and efficient disease diagnosis models. For instance, Machine Learning-Based Disease Diagnosis (MLBDD) systems are designed to streamline diagnostics. In the case of cardiovascular diseases (CVDs), MLBDD systems analyze heart echocardiogram datasets to detect abnormal heart conditions. In biomedical sciences, AI significantly reduces diagnostic errors, thereby minimizing risks during treatment [58,59].

In omics research, the vast and complex datasets generated can be effectively analyzed using AI and machine learning-based tools. These tools are instrumental in interpreting and identifying genomic data, with the goal of improving prognosis, diagnosis, and treatment methods for various disorders. By training ML models to identify promoter sequences, enhancer sequences, splice sites within transcribed RNAs, mutation sites, and genetic variants, researchers can detect patterns of gene expression, particularly in cancer cells [60].

Drug discovery, traditionally a time-intensive and laborious process, involves identifying therapeutic compounds through clinical trials, a process fraught with challenges in predicting drug activity. AI and ML tools have revolutionized this field by expediting the prediction of bioactive compounds’ behavior and properties with high accuracy. By training algorithms on extensive datasets covering toxicity, drug–drug interactions, drug–target interactions, and potential adverse reactions, AI enables the development of personalized medicine tailored to individual patients [61,62,63].

## 11. Diagnostic Artificial Intelligence Tools

Traditional pathological tests for disease diagnosis are often time-consuming, labor-intensive, and require specialized expertise to interpret the results. The integration of AI-based tools has transformed disease detection, making the process more efficient and accessible. Below are examples of automated platforms and tools enhancing diagnostics, which are further described in Table 1.

**Figure 4:**
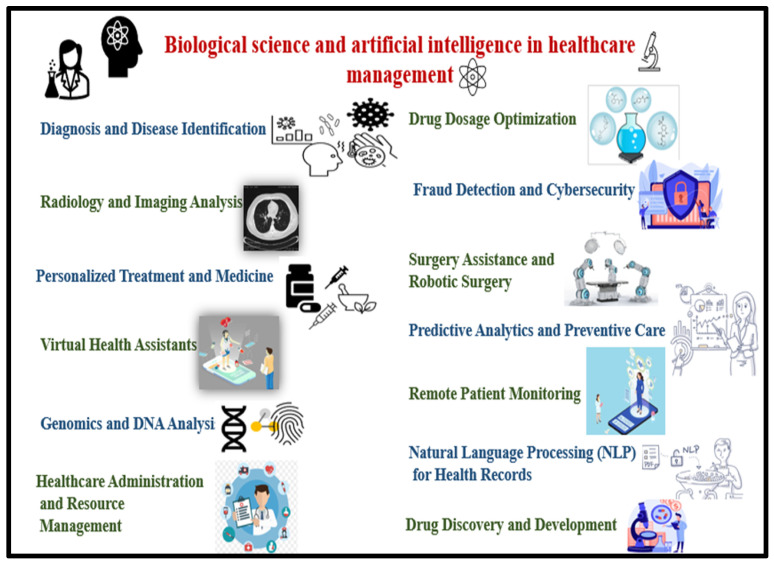
Biological science applications or justifications for artificial intelligence in healthcare management.

- TBDx: This automated smear microscopy system facilitates tuberculosis diagnosis by automating slide preparation and analysis. Prepared slides are placed on the fluorescence microscope stage, magnified at 40×, and digitally imaged. AI algorithms then classify sputum smears as positive or negative by counting acid-fast bacilli (AFB) [64,65]. Although it has demonstrated potential, its diagnostic accuracy in clinical settings fall short as compared to skilled microscopists and advanced diagnostic tests such as Xpert. This could be because TBDx, which relies on image analysis, lacks molecular precision, which results in a higher rate of missed diagnoses and potentially less reliable results. However, in resource-constrained settings, TBDx provides a valuable alternative prioritizing specificity and helping to identify patients who need further confirmatory testing [66]. The feasibility of upscaling TBDx as a diagnostic tool depends on the technological infrastructure of hospitals, such as digital imaging equipment and computing power for data processing. However, the initial expenditure can be substantial. Applications of AI and computer vision in medicine, as shown in Figure 4 highlight their impact on such tools.
- xRapid-Lab and xRapid-Malaria: These iOS-based mobile applications detect malaria using computer vision algorithms to analyze blood smear images obtained through a digital microscope. These tools provide real-time data on parasite counts and red blood cell (RBC) levels, achieving over 98% accuracy [65,67]. However, its accuracy can vary depending on the smartphone camera and lighting conditions. In resource-limited and remote settings, it might face some limitations in terms of scalability as it requires stable electricity, internet connectivity, trained personnel, and seamless integration with existing healthcare systems. But these challenges can be addressed by deploying adaptable, localized solutions fit for the unique needs of each environment, such as using portable solar chargers, an offline local data storage system that is periodically uploaded to the central system, developing simplified, multilingual training modules and peer-to-peer training models, and collaboration with local health authorities for designing compatible data management systems.
- Mobile Apps for Disease Diagnosis: Recent developments include mobile applications for diagnosing and predicting diseases such as COVID-19, cardiovascular diseases, and diabetes. These apps employ machine learning models pre-trained on disease-specific datasets. User inputs are analyzed in real time, with diagnostic results displayed directly on the app [68].
- PCR.AI: This automated tool interprets and analyzes qPCR amplification curves for detecting diseases and pathogens. It offers improved accuracy at reduced costs compared to manual qPCR analysis [69].
- PIVOT: This AI and machine learning-based platform predicts cancer-related genes, such as oncogenes and tumor-suppressor genes (e.g., TP53, PIK3CA, SOX9). Trained on multi-omics datasets—including mutation, gene expression, and variation data—PIVOT identifies genetic abnormalities and provides personalized treatment recommendations for cancer patients [70].

For further details on diagnostic AI tools, see Table 1.

## 12. Active Surveillance and Disease Detection

**Figure 5:**
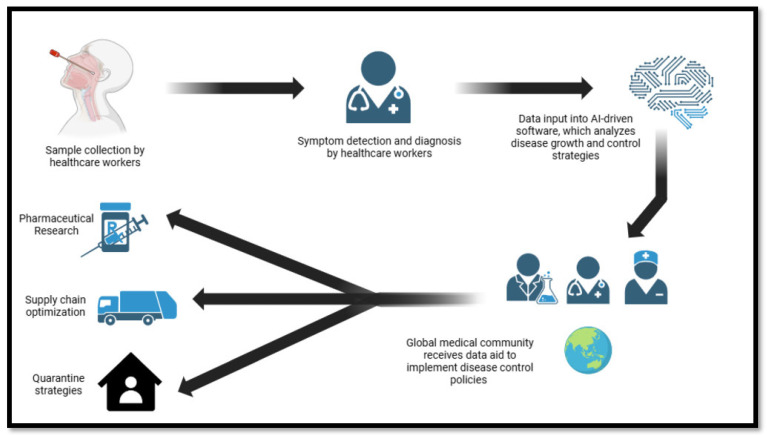
Simplified model of artificial intelligence-based disease control.

The accuracy and reliability of traditional diagnostic methods have been significantly enhanced through AI analysis, leveraging extensive genomic, radiomic, phenomic, and pathogenic databases [71]. AI also offers the potential to reduce dependency on biopsy-based diagnostics, paving the way for non-invasive techniques. By facilitating the development of advanced tools that detect diseases through chemical, vocal, and visual indicators—such as biosensors—AI enhances diagnostic capabilities and supports long-term disease surveillance. These tools help determine prognoses and inform treatment options. Figure 5 provides a simplified model visualizing how AI-based analysis of medical data input can provide rapid solutions to epidemic diseases by assisting in supply chain optimization, creating quarantine and control strategies, and providing analytical assistance in pharmaceutical studies for treatment and prevention. 

Modern biosensors are highly compact and capable of detecting biomarkers from various body fluids, including blood [72], sweat [73], saliva [74], tears [75], and other in vivo sources [76]. Ingestible biosensors integrated into pills can wirelessly collect and transmit patient data to physicians [77,78]. Enhancing these biosensors with machine learning (ML) capabilities would not only improve their sensitivity but also enable the identification of novel biomarkers. AI-driven biosensors provide opportunities for monitoring internal organs, particularly in patients with critical illnesses. This surveillance can track disease progression, such as tumor formation and metastasis, at a chemical level. A biosensor developed by Abbott, known as the Freestyle Libre system, uses AI to predict blood glucose levels. It is a continuous glucose monitoring device that provides real-time data and helps diabetes patients better manage their conditions and optimize treatment regimens [79].

Recent innovations in diagnostic techniques for COVID-19 have accelerated the development of AI-based tools that use vocal biomarkers for diagnosis. These algorithms, in conjunction with behavioral indicators, can also detect neurodegenerative and psychological disorders. However, vocal biomarkers are not limited to these applications. Researchers have identified their presence in various diseases affecting other systems, such as rheumatoid arthritis [80]. For example, a convolutional neural network-based sound classifier demonstrated 90% accuracy in diagnosing COVID-19 through vocal biomarkers [81]. The rapid identification of abnormalities in voice patterns by AI algorithms provides an efficient method for analyzing diseases’ social, epidemiological, and psychological dimensions in a digitalized context.

To diagnose diseases like depression, Parkinson’s disease, and Alzheimer’s disease, algorithms that analyze voice traits, including tone, pitch, and cadence, are being investigated. Before further symptoms appear, modest alterations in speech patterns can be used as early-stage disease markers in neurodegenerative disorders. Based on minute voice changes seen during patient interactions, Zhang et al. demonstrated that a speech-based AI model could identify early Alzheimer’s signs with 90% accuracy [82].

### Genetic Disorders

The use of AI in bioinformatics can help detect and determine the risk of rare genetic disorders in patients. A study testing an ML-based algorithm’s ability to diagnose Disorders of Sexual Development (DSD), a group of genetic congenital disorders, found that the algorithms were able to diagnose cases with an accuracy of 94.1% [83]. Supervised Learning models have shown promising results in diagnosing β-thalassemia in antenatal women in studies held in Vietnam [84], Turkey [85], and India [86].

## 13. Comparison of Traditional and AI-Based Methods in Healthcare Diagnostics

AI has introduced innovative approaches to healthcare, distinct from traditional methods like rule-based systems and manual diagnostics. AI, particularly deep learning models, excels in accurately detecting abnormalities in medical imaging and diagnosing conditions such as cancer, often surpassing radiologists in terms of sensitivity. These systems efficiently manage large datasets and operate continuously, enhancing productivity in high-demand environments, unlike manual diagnostics, which are time-intensive and limited by human availability. AI’s capability to identify subtle patterns in complex data enables predictive modeling for early disease intervention and personalized treatment planning, offering consistent and reproducible outcomes. However, AI’s performance heavily depends on the availability of diverse, high-quality datasets, whereas traditional methods rely on expert knowledge adaptable to incomplete data. The opaque nature of AI, often perceived as a “black box”, contrasts with the transparency of conventional diagnostics based on established procedures. The integration of AI into healthcare requires substantial investment in infrastructure, training, and technology, posing challenges in resource-limited settings where manual methods may be more feasible. Additionally, AI raises concerns about data privacy, accountability for errors, and biases in decision-making. Despite its advantages in accuracy, efficiency, and early detection, AI implementation must be balanced with clinical oversight to address these challenges, necessitating advances in AI explainability and healthcare infrastructure to fully realize its potential. This has been summarized in Table 2.

## 14. Discussion

AI is a rapidly advancing field that has revolutionized biomedical engineering and healthcare in recent years, leading to significant improvements in patient outcomes and the efficiency of healthcare systems. This article reviews studies on AI and its role across various scientific disciplines, including medicine, bioengineering, and healthcare.

Our findings highlight that AI is now applied in numerous subfields of health, such as imaging, diagnosis, segmentation, and even robotic surgery. These advancements are achieved through tools like machine learning, computer vision, and neural networks. Additionally, emerging applications of AI are being developed in areas like gastroenterology, pulmonary diseases, and immunology.

In biomedical engineering, AI addresses complex challenges and enhances patient care. AI algorithms analyze medical images to detect disease patterns, such as early signs of cancer. They also contribute to developing innovative medical devices and technologies, including surgical robots, and facilitate personalized medicine by tailoring treatments to individual genetic profiles.

AI’s impact on healthcare is multifaceted. It improves medical devices and technologies, enhances patient outcomes, and reduces healthcare costs. One crucial application is the identification and prediction of disease development, enabling early intervention and prevention. For instance, AI automates tasks like maintaining medical records, scheduling appointments, analyzing health data, and streamlining healthcare system operations.

The other real-world application of AI would include predicting emerging and chronic diseases based on the past, biomarkers, imaging, etc. This ability is especially needed in countries where so much healthcare funding is spent on chronic diseases that can be prevented, such as diabetes, cardiovascular disease, and cancer. These diseases can be predicted and shortened through AI, both for patients and healthcare systems.

A major challenge to AI implementation is its accessibility. AI-driven healthcare solutions must be equally beneficial to all sections of society regardless of socio-economic (economic background, gender, culture) and biological (sex, race, ethnicity) variables. The major factors influencing this issue include the affordability and economic viability of AI-driven technology in healthcare, as well as AI bias. The former must be considered to prevent the accessibility of AI-based healthcare to concentrate solely to those in the higher socio-economic strata. The former can arise out of human prejudice, as the data on which AI is trained can often contain human bias. It can affect the quality of services provided to different patients, which is undoubtedly a major ethical concern.

Several other ethical hurdles come forward when discussing the role of AI in healthcare. The legal framework defining the responsibilities of AI use, and regulating its misuse, is still at a nascent stage, leading to a vast ‘gray zone’ as the capabilities of AI progress. Such issues can arise when facing issues such as bias. Another area where regulatory policies can clash with AI development is data privacy and informed consent laws. While strict data confidentiality legislations may protect patient data, they hinder AI training and development in healthcare applications. On the other hand, tax regulations may infringe upon patient confidentiality and rights.

Moreover, while AI can analyze vast amounts of data, it still struggles to fully grasp the nuanced medical knowledge necessary for accurate diagnosis and treatment, especially when it comes to understanding the context of a patient’s condition. The integration of AI into healthcare brings up important ethical questions, particularly about accountability and informed consent [87]. Patients must be kept in the loop about how their data is being used, with their consent sought regularly, and there needs to be open discussion about how AI affects their care. One pressing issue is figuring out who should be held responsible if an AI system makes a mistake, whether it is the developers, the healthcare providers, or someone else.

This highlights the importance of creating a system where AI supports human expertise rather than replaces it. AI can certainly assist in clinical decision-making, but it is essential that doctors stay involved to interpret results within the broader context of the patient’s life and values. There is also the risk that AI could reinforce existing biases or even create new inequalities if it is trained on biased or incomplete data. Developers need to ensure that their datasets are diverse and representative to prevent such outcomes. To tackle these issues, we need stronger rules for data management and clear guidelines to protect patient privacy. Creating AI models that are easier to understand is also crucial, as this would help healthcare professionals trust the system and work with it more effectively. By making AI more transparent and ensuring that it works alongside doctors, we can build trust and maximize its potential to improve healthcare.

As AI systems rely heavily on large, high-quality datasets, these systems might face challenges in low-resource settings, particularly in rural areas. This necessitates the optimization of AI technologies that can adapt to such limitations. In settings with limited patient data, AI models can be trained using data augmentation techniques to artificially expand datasets and improve model robustness. Moreover, AI models can be optimized to run on low-power devices, such as smartphones or tablets, and adopting edge computing can make AI more accessible to healthcare workers in rural areas. Integrating AI-based diagnostic tools with voice commands and visual interfaces could make it easier to navigate for healthcare workers with limited technical knowledge.

AI can be used in disease surveillance and to predict disease outbreaks. Future research could focus on developing AI tools that can be integrated with remote sensing technologies, such as satellite imaging to track disease conducive environmental conditions. Another potential field of development is AI integrated telemedicine, where AI can assist in triaging patient cases, determining the urgency of conditions, and providing initial diagnostic support. This can help bridge the gap between rural patients and healthcare professionals. Moreover, it can be a useful tool for optimizing healthcare resource allocation by predicting healthcare needs based on historical data and seasonal and current disease patterns.

## 15. Conclusions

The application of AI has proven to be highly promising in many aspects of healthcare. It also helps clinicians and patients, making the diagnosis, decision-making, treatment, and surgery of disease simpler. In addition, AI reduces hospital bills that are not always accessible to patients.

But access to care is still skewed, especially in poorer neighborhoods far from cities, where hospitals and medical personnel tend to be stacked together. Such a difference could be overcome through the development of AI-based health systems that are simple, affordable, and readily available. This kind of system would help the world to fulfil the Sustainable Development Goal of promoting healthy living for all.

AI’s potential to prevent disease is especially exciting. Through early detection and forecasting, AI can prolong lives, decrease the rate of illness, and avoid death. These discoveries can also help patients pay less for healthcare in the long term by preventing diseases rather than treating them.

AI could transform biomedical engineering and medicine by optimizing medical devices, technologies, outcomes, and efficiency at a fraction of the cost. AI has some limitations, but algorithmic and technical advancements will soon fix these issues and AI systems will be safe, trustworthy, and morally designed.

## Figures and Tables

**Table 1:** Diagnostic artificial intelligence tools.

AI Tools	Disease/Diagnosis	Features
TBDx	Tuberculosis	Automated smear microscopy system; count the number of acid-fast bacilli; classify smears as positives or negatives.
xRapid-Lab and xRapid-Malaria	Malaria	Automated malarial parasite and red blood cell counter; determine different developmental stages of the parasite.
PCR.AI	qPCR analysis	Automated interpretation and analysis of qPCR.
PIVOT	Cancer	Detection and prediction of cancer-causing genes in cancer patients.

**Table 2:** Comparison of traditional versus AI-based methods in healthcare diagnostics.

Traditional Methods	AI-Based Methods
People had to write strict rules and instructions for tasks.	AI learns on its own from patterns in data.
These methods could only handle specific, predefined tasks.	AI adapts to a wide variety of problems and keeps learning.
A lot of human effort was needed to update or fix things.	AI can automate tasks and needs less human involvement after training.
Worked well with small, organized datasets, like spreadsheets.	Can handle huge, messy datasets, including images, videos, and text.
Accuracy was often low, especially for complex problems.	AI is highly accurate and keeps getting better as it learns more.
Processing large amounts of data was slow and time-consuming.	AI processes data much faster and can even work in real time.
Translating languages or searching for keywords relied on manual work.	AI handles translations and understands human language much better.
Recognizing images required rigid rules.	AI can classify images and even recognize faces or objects seamlessly.
Problems were solved step-by-step, following fixed logic.	AI thinks more like a human brain, using probabilities and creativity.
Any changes required for manual reprogramming.	AI evolves and improves automatically with new data.
Humans had to perform repetitive tasks over and over.	AI takes over boring tasks, so people can focus on creative work.
Errors were hard to spot and fix without human help.	AI can spot and correct its own mistakes as it learns.
Adapting to new situations was difficult and slow.	AI adjusts quickly to changing conditions and environments.
It seemed cheaper at first but became costly in the long run.	AI might cost more upfront, but it saves money over time by being efficient.
Doctors relied on experience and manual checks for diagnoses.	AI assists doctors with faster, more accurate diagnoses and treatments.
Research was slow, relying on manual data collection and analysis.	AI accelerates research with predictions, insights, and automated tools.
